# Review on 2D Molybdenum Diselenide (MoSe_2_) and Its Hybrids for Green Hydrogen (H_2_) Generation Applications

**DOI:** 10.1021/acsomega.2c00330

**Published:** 2022-05-12

**Authors:** Muhammad B. Wazir, Muhammad Daud, Soma Safeer, Faisal Almarzooqi, Ahsanulhaq Qurashi

**Affiliations:** † Department of Chemical Engineering, Khalifa University of Science and Technology, Main Campus, 127788 Abu Dhabi, United Arab Emirates; ‡ Department of Chemical Engineering, University of Engineering and Technology, 25120 Peshawar, Pakistan; § Department of Chemistry, Khalifa University of Science and Technology, Main Campus, 127788 Abu Dhabi, United Arab Emirates

## Abstract

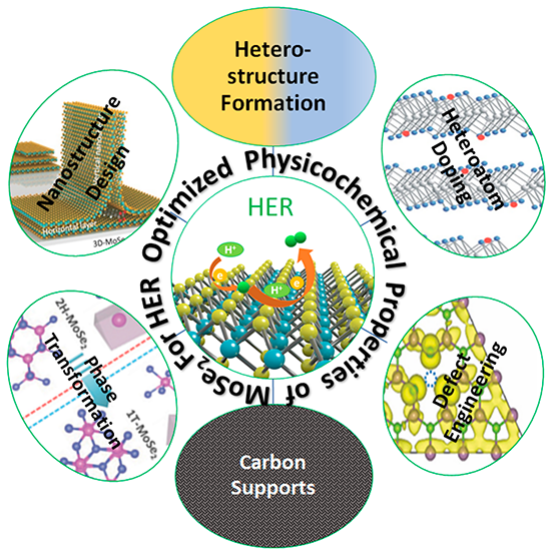

Hydrogen (H_2_) is a green and economical substitute to traditional fossil fuels due to zero carbon emissions. Water splitting technology is developing at a rapid speed to sustainably generate H_2_ through electro- and photolysis of water without the harmful emissions associated with steam methane reforming. Development of efficient catalysts for the hydrogen evolution reaction (HER) is pertinent for economical green H_2_ generation. In this regard, 2D transition metal dichalcogenides (TMDCs) are considered to be excellent alternatives to noble metal catalysts. Among other TMDCs, 2D MoSe_2_ is preferred due to the low Gibbs free energy for hydrogen adsorption, good electrical conductivity, and more metallic nature. Moreover, the physicochemical and electronic properties of MoSe_2_ can be easily tailored to suit HER application by simple synthetic strategies. Herein, we comprehensively review the application of 2D MoSe_2_ in the electrocatalytic HER, focusing on recent advancements in the modulation of the MoSe_2_ properties through nanostructure design, phase transformation, defect engineering, doping, and formation of heterostructures. We also discuss the role of 2D MoSe_2_ as a cocatalyst in the photocatalytic HER. The article concludes with a synopsis of current progress and prospective future trends.

## 1 Introduction

The excessive reliance on conventional fossil fuels and the exponentially increasing demand for energy is leading to a rapid depletion of these resources with increased carbon emissions proving detrimental for the environment. The current scenario poses an imperious need for alternate (green and economical) energy technologies to best fulfill these demands. Green hydrogen is a reliable substitute for traditional fossil fuels owing to its characteristics of zero carbon emissions, recyclability, high conversion efficiency, and high energy density. Hydrogen is predicted to be supplying 11% of the total energy demands of the globe by 2025 and 34% by 2050.^1^

Currently, hydrogen is produced on the industrial level by steam methane reforming of natural gas, resulting in carbon dioxide emissions. However, electro- and photocatalytic water splitting are preferred, as renewable energy sources are used in the production of hydrogen.^2^ Precious noble metals such as ruthenium (Ru), iridium (Ir), palladium (Pd), platinum (Pt), and their alloys are the most efficient catalysts for electrocatalytic and photocatalytic water splitting owing to their optimum absorption and binding energies for hydrogen and protons. However, natural scarcity and consequently the high cost of such metals limit their application.^3^ Recently, transition metal-based materials have gained the attention as efficient HER catalysts.^4^ Among these, two-dimensional (2D) earth-abundant transition metal dichalcogenides (TMDCs) are preferred due to their tunable characteristics.^5−7^

MoS_2_ has been extensively investigated as an effective HER catalyst. However, experimental studies have shown MoSe_2_ to be more favorable due to its more metallic nature, lower Gibbs free energy, and narrower band gap.^8−10^ The catalytic activity of MoSe_2_ greatly depends on the edge sites and its morphology.^8^ In addition, the small band gap and ability to act as an electron exporter in bicatalytic systems make MoSe_2_ a reliable cocatalyst for photolysis of water.^8,11^ Nevertheless, drawbacks like poor conductivity relative to noble metal catalysts and aggregation of MoSe_2_ during fabrication are pushing researchers to introduce novel techniques to further improve the HER performance of MoSe_2_.^3^

**Scheme 1:**
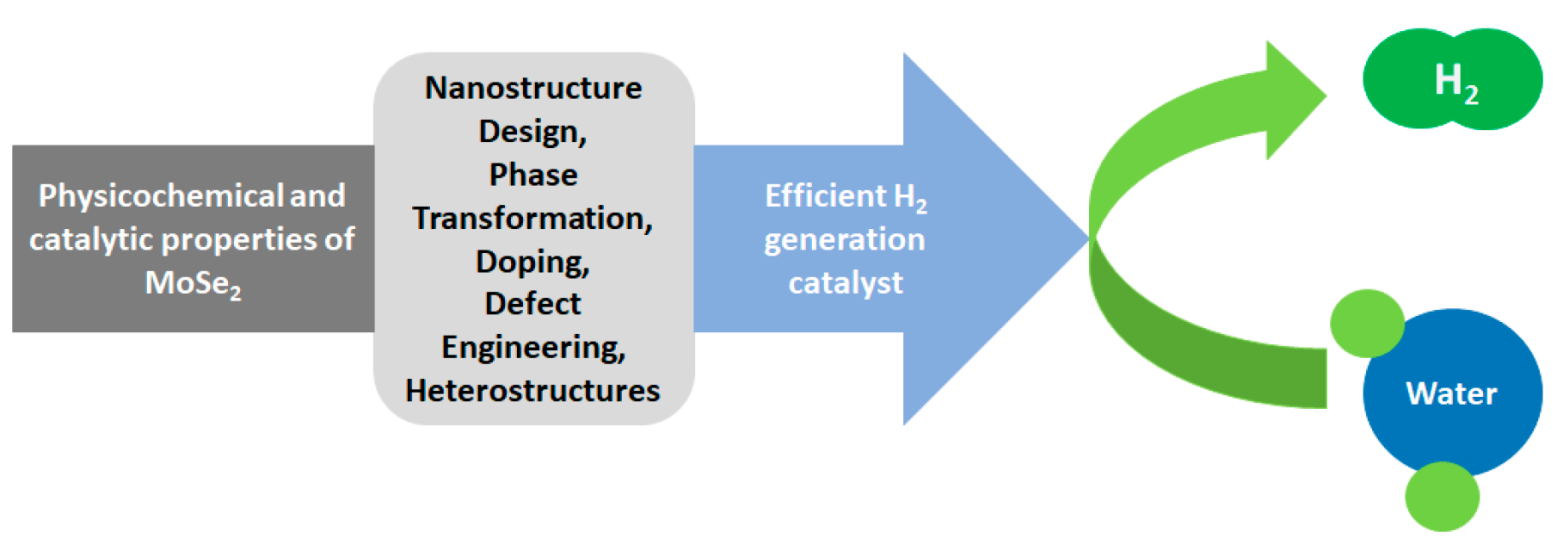
Conceptual Illustration of Tuning the MoSe_2_ Properties for the HER

A few review articles are available on closely related topics. For instance, Xia et al.^3^ reviewed the progress of transition metal selenides for HER electrolysis. Recently, Nithya studied the recent progress in CoSe_2_-based electrocatalysts for H_2_ generation.^12^ Another review article presented the role of various active sites and ways to increase active sites in metal sulfides.^13^ The design, synthesis, property modulation, and mechanisms of 2D transition metal dichalcogenide-based electrocatalysts have also been reviewed.^10^ However, there is no review article hitherto which comprehensively focuses on the application of 2D MoSe_2_ nanocatalysts for hydrogen generation. Herein, we will provide an up to date review of the current literature on strategies to enhance the HER activity of MoSe_2_-based materials. We will elucidate the rationale and effects of modulating the catalytic and physicochemical properties of MoSe_2_ through nanostructure design, phase transformation, heteroatom doping, defect engineering, and heterostructure formation as depicted in Scheme 1.

## 2 Structure and Properties of MoSe_2_

MoSe_2_ has a two-dimensional (2D) lattice structure like MoS_2_ in which a single-layered nanostructure is comprised of a layer of Mo atoms sandwiched between two layers of Se atoms. A few-layered structure is formed by the weak van der Waals interlayer interaction among several monolayers.^14^ MoSe_2_ may exist as semiconductive 2H phase with trigonal prismatic geometry or a metallic 1T phase with octahedral geometry.^15^ A small band gap makes MoSe_2_ relevant for photoelectrochemical applications.^3^ The low Gibbs free energy of MoSe_2_ for hydrogen adsorption makes it a promising catalyst for the HER. MoSe_2_ also exhibits higher electrical conductivity than MoS_2_ owing to the more metallic nature of Se (1 × 10^–3^ S m^–1^) than S (5 × 10^–28^ S m^–1^).^5^ In addition, the 1T phase exhibits higher electrical conductivity than the 2H phase. However, the metastable metallic 1T phase is thermodynamically unstable and can readily be converted into the 2H phase.^15,16^ In the following sections we will elucidate the recent approaches to tailor the properties of MoSe_2_ for enhanced HER performance beyond intrinsic levels.

## 3 Modulating the Electrocatalytic and Physicochemical Properties of Pristine MoSe_2_ for Enhanced Hydrogen Generation

### 3.1 Nanostructure Design

**Figure 1:**
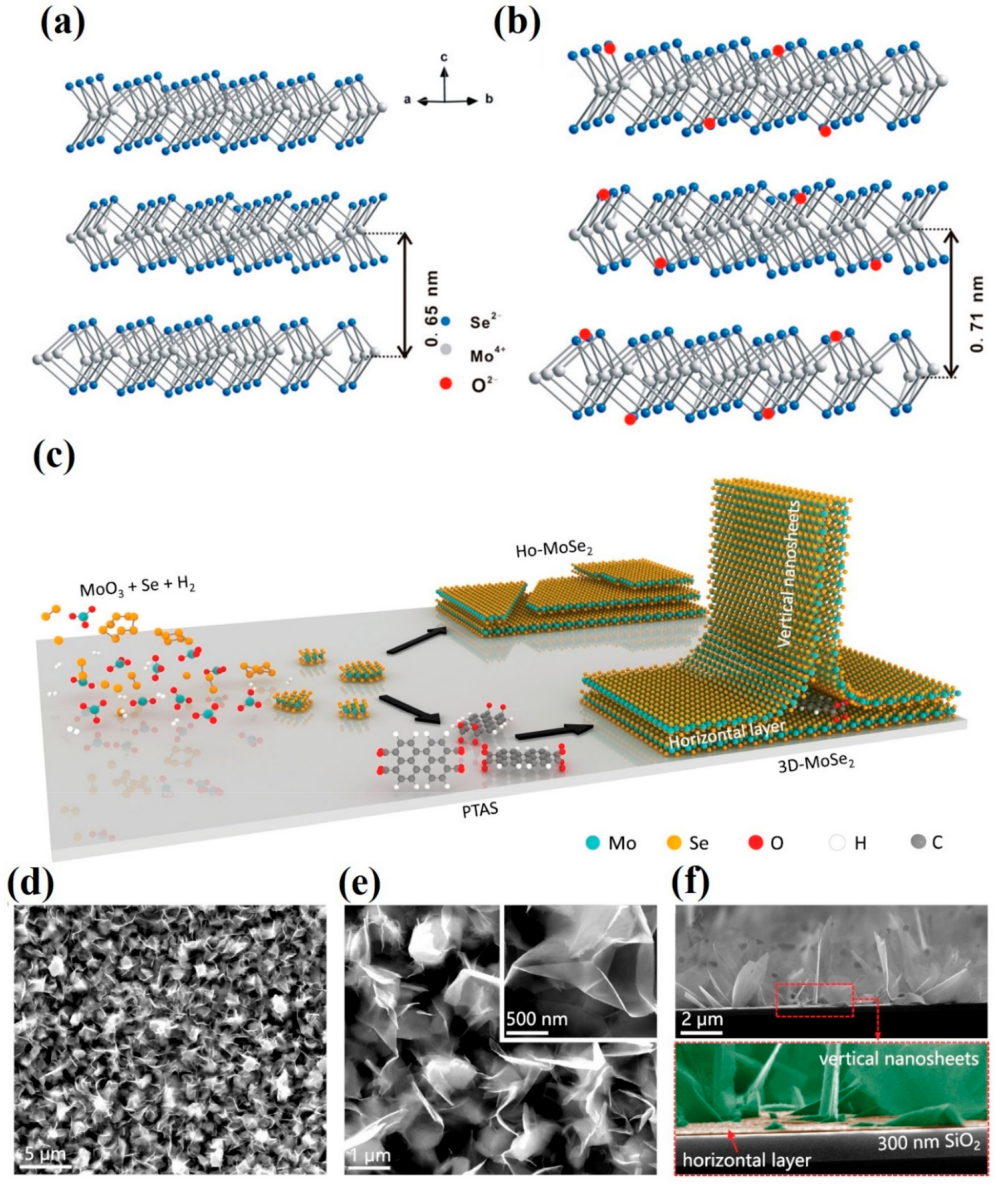
Nanostructure design of 2D MoSe_2_. (a) Pristine MoSe_2_. (b) Interlayer expansion of MoSe_2_ by oxygen incorporation. Reprinted with permission from ref (14). Copyright 2017 American Chemical Society. (c) Schematic illustration of MoSe_2_ nanoarchitecture synthesis with the help of PTAS as a seed promoter. SEM images of PTAS-assisted grown 3D MoSe_2_: (d and e) top and (f) side views at different magnifications. Reprinted with permission from ref (17). Copyright 2017 IOP Publishing.

The fabrication of nanostructures is an excellent way of improving the electrocatalytic performance of 2D MoSe_2_ as it exposes abundant active sites. However, 2D architectures of MoSe_2_ (e.g., nanosheets and nanoflakes) often need a substrate support to be employed as an electrode in the electrocatalytic HER. The perpendicular stacking of 2D MoSe_2_ nanosheets on a conductive support is a favorable nanoarchitecture, which can promote the HER by giving access to abundant active sites and improving the charge transport. Dai et al.^14^ used the rGO platform to disperse defect-rich oxygen-incorporated MoSe_2_ nanosheets with an interlayer spacing of 0.71 nm by the PVP-assisted hydrothermal method as illustrated in Figure 1a and 1b. The rGO helped to improve the charge transport due to good electrical conductivity and acted as a platform to disperse MoSe_2_ nanosheets to maximize exposed sites. Therefore, the resultant 2D nanosheets supported on interconnected conducting network exhibited improved HER performance.

Efforts to improve the HER activity by designing a 2D nanoarchitecture in the form of nanosheets and nanoflakes and modulating their electronic and catalytic properties have surely produced promising results. However, to further improve the electrocatalytic performance, these 2D structures are extended in three-dimensional space. An increased packing density of active sites can be obtained by merging the active-site-rich 2D nanosheets in a 3D space. It not only provides access to more active sites but also improves the electronic conductivity through interconnected networks. A highly crystalline 3D hierarchical nanoarchitecture composed of few-layered perpendicular MoSe_2_ nanosheets anchored onto the 2D MoSe_2_ horizontal layer was constructed through the chemical vapor deposition method by employing perylene-3,4,9,10-tetracarboxylic acid tetrapotassium salt (PTAS) as a seeding agent as illustrated in Figure 1c. The corrugated ultrathin nanosheets with an average width and height of 1.69 and 2.74 μm, respectively, were grown vertically onto the horizontal 2D nanosheet (Figure 1d–f). The as-obtained 3D architecture exhibited less charge transfer resistance (only 2%) and more electrochemically active surface area (∼12 times) than the 2D MoSe_2_ layer. The Tafel slope of 3D MoSe_2_ was reduced from 123.8 to 47.3 mV dec^–1^.^17^ The 3D flower-like nanoarchitectures constructed by superposition of 2D nanosheets are also preferred due to the plethora of exposed edge sites, higher surface areas, and improved electrical conductivity. Tran et al.^18^ prepared porous MoSe_2_ nanoflowers with enhanced morphology by selective etching of copper (Cu) from MoSe_2_@Cu_2_Se. The stacked 2D-MoSe_2_ nanosheets, emulating the shape of petals, with a more open structure not only led to the proliferation of active sites but also boosted the conductivity, greatly enhancing the HER activity of porous rose-like MoSe_2_ nanostructures.^18^

However, to fully exploit the HER potential of MoSe_2_ nanostructure design is usually coupled with other physicochemical property modulation techniques including phase transformation, doping, defect engineering, and heterostructure formation. Table 1 compares the performance of MoSe_2_ before and after modification to highlight the extent and mechanism of the modulation effect.

**Table 1:** Performance Comparison of MoSe_2_-Based Catalysts for HER and Underlying Mechanisms

		HER			
catalyst	current density (mA/cm^2^)	overpotential (mV)	Tafel slope (mV/dec)	underlying mechanism for performance enhancement	ref
interlayer-expanded 1T-MoSe_2_	10	179	78	2H → 1T phase transformation and layer expansion resulting in a lower Gibbs free energy for H adsorption/desorption and anincreased number of active sites	(19)
2H-MoSe_2_	10	558	141		
plasma	10	148	51.6	more optimized active sites owing to Se vacancies and holes created through etching	(20)
etched MoSe_2_ (at 20 W)					
1T-MoSe_2_ MoSe_2_-4-180 (MoSe_2_-*x*-*T*, *x* = NaBH_4_: Na_2_MoO_4_·2H_2_O, *T* = temperature °C)	10	152	52	2H → 1T phase transformation coupled with defect formation leading to increased intrinsic activity and more unsaturated Se active sites, respectively	(15)
MoSe_2_-1-180	10	355	146		
MoSe_2_-4-140	10	211	72		
MoSe_2_-4-160	10	197	54		
MoSe_2_-4-200	10	163	55		
Pt			30		
defect-rich exfoliated MoSe_2_	10	350	90	structural defects, multiple Se vacancies, Mo_Se_ antisite point defect, etc.	(21)
bulk MoSe_2_			150		
Ni-doped MoSe_2_	10	184	83	Ni–dopant-induced active sites and lower charge transfer resistance	(22)
MoSe_2_ nanosheets	10	335	118		
MoSe_2_/N-doped carbon	10	142	62	optimized adsorption and desorption of H* due to N-doped carbon confinement	(23)
MoSe_2_	10	468	164		
N-doped carbon	10	859	286		
Pt/C	10	33	34		
MoSe_2_–NiSe epitaxial growth strategy	10	210	56	synergistic interaction between MoSe_2_ and NiSe and enhanced conductivity	(7)
	1	160	TOF: 5.6 s^–1^ at 250 mV		
pure MoSe_2_	10	278	95		
MoSe_2_ + NiSe mixture	10	299	74		
MoSe_2_/MoO_2_/Mo	10	142	48.9	synergistic effect of combining abundant active sites of MoSe_2_ and improved conductivity across MoSe_2_/MoO_2_/Mo	(24)
MoSe_2_/Mo	10	267	65.2		
MoSe_2_/WS_2_	10	75	60	rapid interface charge transport and increased edge active sites	(25)
MoSe_2_	10	112	136		
WS_2_	10	158	114		

### 3.2 Phase Transformation

**Figure 2:**
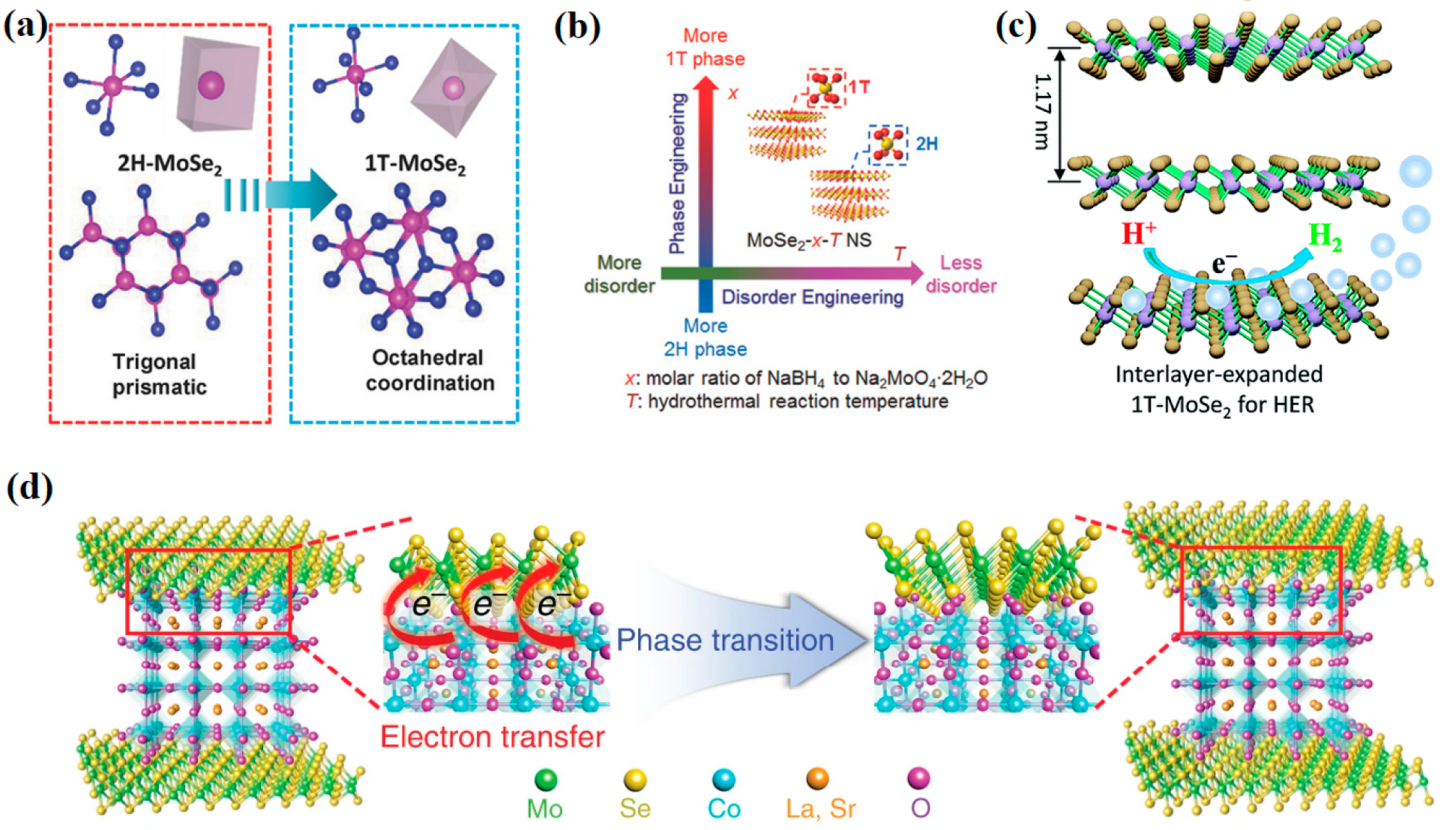
2H → 1T phase transformation of 2D MoSe_2_. (a) Schematic illustration of the crystal structure of 2H- and 1T-phase MoSe_2_. Reprinted with permission from ref (16). Copyright 2018 Wiley. (b) Schematic description of disorder and phase and engineering in 2D MoSe_2_ through hydrothermal synthesis by regulating the reaction temperature (*T*) and ratio of NaBH_4_ to Na_2_MoO_4_·2H_2_O (*x*). Reprinted with permission from ref (15). Copyright 2017 Wiley. (c) Graphical depiction of the HER activity of interlayer-expanded 1T MoSe_2_. Reprinted with permission from ref (19). Copyright 2016 Royal Society of Chemistry. (d) Schematic illustration of in situ 2H → 1T phase transition in MoSe_2_ through spontaneous electron transfer from Co to Mo. Reprinted with permission from ref (27). Copyright 2019 Nature.

Phase transformation of 2D MoSe_2_ from a typical p-type semiconducting 2H phase with a trigonal prismatic lattice to a metallic 1T phase with a trigonal octahedral lattice (Figure 2a) promotes electrical conductivity and activates basal planes to improve the HER performance.^16^ Ambrosi et al.^26^ chemically exfoliated MoSe_2_, MoS_2_, WSe_2_, and WS_2_ using the Li intercalation method. A more efficient and effective 2H → 1T phase transformation was achieved in MoSe_2_ and WS_2_ as compared to other counterparts. Noticeably, the 2D MoSe_2_ showed a 300 mV shift in overpotential and the best overall HER performance with the lowest Tafel slope after exfoliation.

The metastable metallic 1T phase of MoSe_2_ can readily be converted into 2H phase, indicating lower stability.^28^ The integration of metastable 1T phase with 2H phase is often utilized to prepare stable mixed 1T/2H phase 2D MoSe_2_ for improved HER activity.^19^ For instance, Xiao et al.^29^ prepared stable multiphase 1T/2H-MoSe_2_ by tuning the amount of NaBH_4_ reductant. The severe reduction process at excess NaBH_4_ concentration can lead to electronic structure change, conducive to 2H → 1T rearrangement, in the MoSe_2_ framework.^15^ The synergistic combination of phase transformation with nanostructure formation and doping have also resulted in improved performance for the electrocatalytic HER.^16^ For instance, Yin et al.^15^ synergistically coupled the disorder engineering with phase transition by regulating the temperature and the amount of NaBH_4_ in hydrothermal synthesis (Figure 2b). Jiang et al.^19^ integrated the phase transition with interlayer spacing (Figure 2c) to benefit from additional sites provided by defect-rich mixed 1T and 2H phases, increased density of exposed sites, optimized hydrogen adsorption free energy, and enhanced electronic conductivity of metallic 1T phase. Recently, Oh et al.^27^ reported an in situ 2H → 1T phase transition in MoSe_2_ during the formation of the heterostructure with perovskite oxide La_0.5_Sr_0.5_CoO_3−δ_ (LSC) induced by spontaneous electron transfer from Co to Mo as illustrated in Figure 2d. The phase transition was induced by alteration of the electronic configuration of the Mo 4d orbital from occupied 4d_*z*^*2*^_ to incompletely filled d_*xz*_, d_*yz*_, and d_*yz*_ due to additional electrons.^27^

### 3.3 Defect Engineering

**Figure 3:**
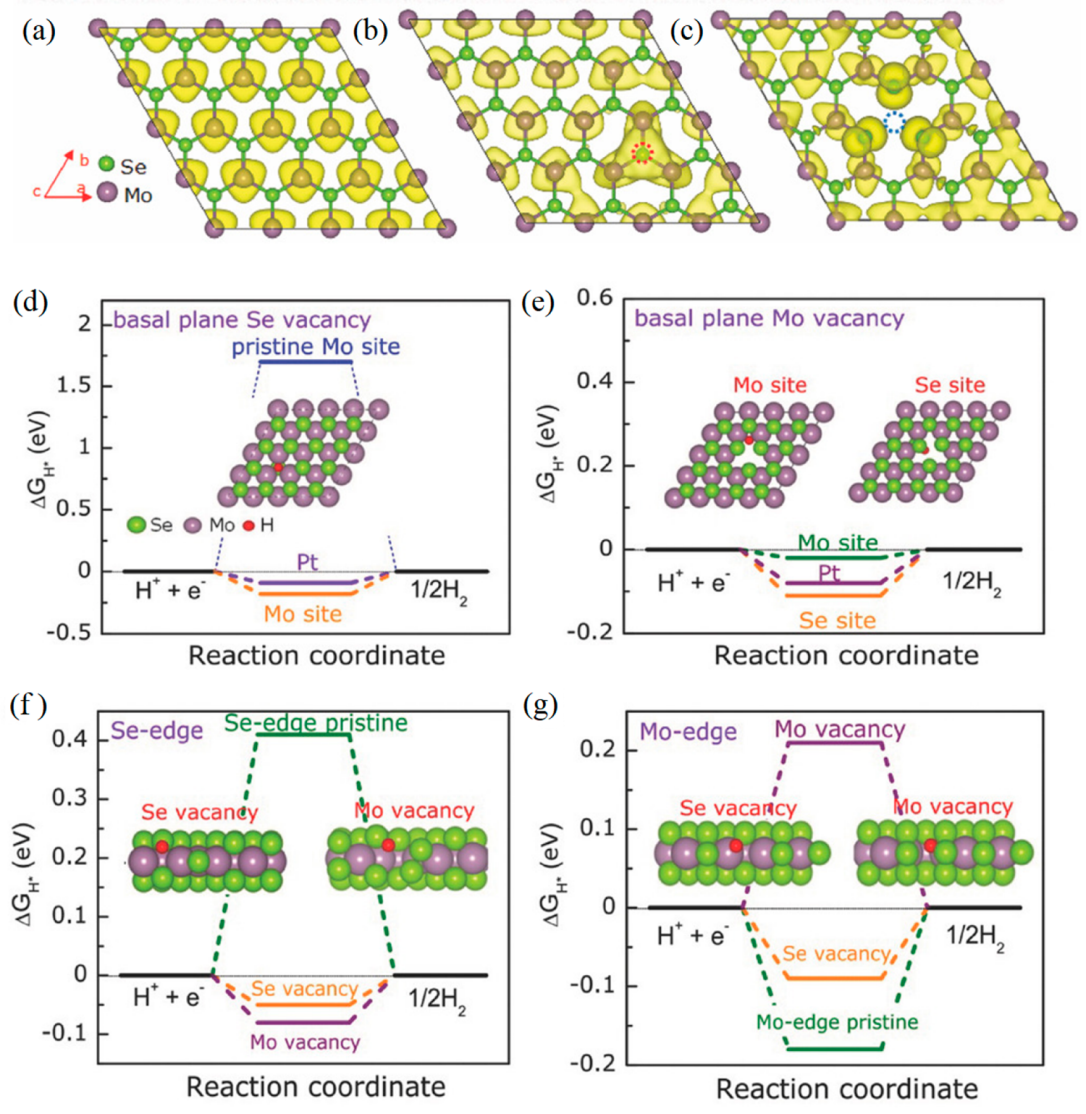
Vacancy-induced activation of the basal plane of 2D MoSe_2_. Partial charge densities of 2D MoSe_2_: (a) pristine, (b) 3.12 atom % Se vacancy, and (c) 6.12 atom % Mo vacancy. Gibbs free energy for hydrogen adsorption at the Mo site and Se site for (d) basal plane Se vacancy, (e) basal plane Mo vacancy, (f) Se edge with Se vacancy, and (g) Mo edge with Mo vacancy. Reprinted with permission from ref (6). Copyright 2018 Wiley.

In pristine 2D MoSe_2_, only the edge sites are active for catalytic H_2_ evolution. The enlargement of the MoSe_2_ edge thorough pore engineering enhances the HER performance to some extent. However, to fully utilize the large proportion of stable basal planes of 2D TMDCs, defect engineering is often employed to activate these passive basal planes for H_2_ evolution.^30^ The defects, including chalcogen vacancy, metal vacancy, line, and antisite, etc., result in alteration of the electronic structure and modify the intrinsic characteristics of 2D MoSe_2_ in favor of electrocatalytic H_2_ generation.^6,20,21^ For instance, vacancy creation induces the catalytic activity in the inert basal plane and edges of 2D MoSe_2_ and enhances the electronic conductivity.^6,20^ Gao et al.^6^ produced dual Mo and Se vacancies in 2H MoSe_2_ through chemical vapor deposition. These vacancies induced active catalytic sites in the basal plane and on the edges by reducing the energy barrier of MoSe_2_ for H^+^ adsorption. These vacancies also facilitated the electron transport by boosting the number of electrons and gap states near the Fermi level for efficient electrocatalytic HER. The alterations in charge densities of potential active sites created by Se and Mo vacancies can be observed in Figure 3a–c. Furthermore, Figure 3d and 3e illustrates the low Gibbs free energy of H^+^ adsorption at Mo and Se vacancy sites on the edge and basal plane, indicting activation of the inert basal plane by dual vacancies.^6^ Similarly, Truong et al.^21^ prepared exfoliated MoSe_2_ sheets with multiple defects, favoring catalytic H_2_ evolution, including Se vacancy, Se_2_ vacancy, MoSe antisite, and adatoms through a supercritical fluid process. A recent study accompanied by finite element and first-principles density functional theory calculations also confirmed that vacancies create additional active sites by reducing Δ*G*_H*_ and improve the electrical conductivity by decreasing the band gap.^20^Table S1 presents the Gibbs free energy at Mo and Se sites for various vacancies. In conclusion, engineered structural defects tailor the physicochemical properties of 2D MoSe_2_ for HER activity beyond intrinsic levels.

### 3.4 Heteroatom Doping

Heteroatom doping is another way to activate the inactive basal planes of MoSe_2_. Doping of metal and nonmetal heteroatoms in 2D MoSe_2_ alters the electronic structure and modulates the density of states at the Fermi level. This, in turn, activates the basal planes and inactive edges of 2D MoSe_2_ for electrocatalytic HER. Gao et al.^31^ activated the basal planes and Se edge of 2D MoSe_2_ by B doping. The first-principles calculations suggested an improvement in the catalytic activity of MoSe_2_ for H_2_ evolution upon substitution of Se by B. This was experimentally confirmed, as B-doped MoSe_2_ exhibited a lower overpotential, reduced Tafel slope, and five times higher TOF than pristine MoSe_2_. Although Se sites at the basal plane of B-doped MoSe_2_ were found to be inactive for the HER with Δ*G*_H*_ ≈ 1 eV, the B sites were active for the HER with Δ*G*_H*_ values of −0.15 and 0.05 for one and two B-atom doping, respectively. In addition, B doping activated the Se sites at both the Mo and the Se edges by lowering the Δ*G*_H*_ to near zero.

**Figure 4:**
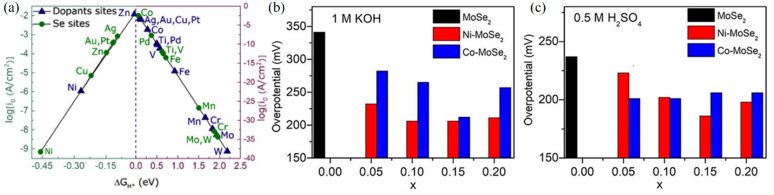
Doped MoSe_2_ for electrocatalytic HER. (a) Calculated volcano plot of MoSe_2_ doped with different transition metals. Reprinted with permission from ref (32). Copyright 2019 Elsevier. Electrocatalytic HER performance of Ni- and Co-doped MoSe_2_ in (b) alkaline and (c) acidic media. Reproduced with permission from ref (33). Copyright 2019 Wiley.

Metal doping in 2D MoSe_2_ has a similar effect to nonmetal doping in terms of increasing the active sites and improving the electrical conductivity. Qian et al.^32^ plotted the Gibbs free energy of H^+^ adsorption on metal dopant sites and adjacent Se sites in a volcano plot to identify potential dopants (Figure 4a). Notably, Zn showed potential for both acting as an active site and activating adjacent Se sites. Zhao et al.^33^ successfully utilized the transition metal (Ni, Co)-doped MoSe_2_ for enhanced electrocatalytic HER in both acidic and alkaline media as illustrated in Figure 4b and 4c. Similarly, Yang et al.^22^ reported Ni–MoSe_2_ for hydrogen evolution in alkaline medium with improved stability owing to the formation of Ni–Se bonds, keeping Ni dopants intact after a long-term cycling process.

### 3.5 Carbon Support

The conductive support is essentially important to fully exploit the superior HER potential of 2D MoSe_2_. Carbon supports provide a platform for electrocatalyst dispersion, electrical conductivity, better electrocatalytic properties, high stability, energetic transfer of an electron at the catalyst–support interface, and abundant redox reaction sites. Highly conductive carbon-based materials provide excellent support for the growth of MoSe_2_ nanostructures. Fabricating MoSe_2_ nanoarchitectures on carbon substrates enhances charge transport, and the hybrid inherits the intrinsic stability and conductivity of the substrate. For instance, the HER performance of the MoSe_2_ electrocatalyst was significantly enhanced by the flexibility and strength provided by a carbon cloth.^34^ Similarly, a hybrid with an enhanced morphological structure was obtained by combining MoSe_2_ with graphene oxide, which aided the electrocatalytic kinetics at the interface and also lowered the electron transfer resistance.^35^ The core–shell C@MoSe_2_ hybrid with a conductive carbon core prevented agglomeration and restacking of MoSe_2_ nanosheets and reduced the charge transfer resistance by providing electrical contact between the MoSe_2_ shells.^36^ Even though these support materials might not actively take part in the electrolysis, conductive supports greatly amend the HER performance of MoSe_2_ by ameliorating the surface morphology, nanosheet distribution, agglomeration, and electrical conductivity.

To take the role of carbon-based supports in enhancing the HER activity of carbon-supported MoSe_2_ a step further, carbon-based materials can also be activated through heteroatom doping.^23,37^ Nitrogen-doped carbonaceous materials show superior HER activity as N doping lowers the Gibbs free energy for H^+^ adsorption in carbon materials like carbon shells.^23^

### 3.6 Heterostructure Formation

Heterostructures of MoSe_2_ are formed with other electrocatalytically active materials to benefit from synergistic combination of the physicochemical, electronic, and morphological properties.^25^ Heterostructure formation with other metallic materials greatly enhances the electrical conductivity of the MoSe_2_ hybrid by providing additional channels for charge transportation. In addition, the hybrid material benefits from the synergistic combination of physicochemical properties of all of the constituents. Yang et al.^38^ integrated MoSe_2_ with Bi_2_Se_3_ to obtain a hybrid with enhanced charge transfer properties and a lower Tafel slope approaching that of noble metals. Moreover, integrating MoSe_2_ with catalysts exhibiting a similar crystallographic structure and lower lattice mismatch results in formation of well-defined heterointerfaces with efficient charge transport.^7,39^ For instance, MoS_2_ and hexagonal NiSe exhibit a lower lattice mismatch of 3% and 10.17%, respectively. Combining MoSe_2_ with MoS_2_^39^ and NiSe^7^ resulted in epitaxial growth of nanocrystallites with active heterojunctions exhibiting a larger surface area and improved charge transport. Likewise, core@shell heterostructures also improve the interfacial charge transfer resulting in enhanced HER performance.^40^ Defect formation at the heterointerface contributes to formation of additional active sites and activates the basal planes of MoSe_2_. Intercalation of 2D MoSe_2_ nanosheets with porous CoP sheets not only resulted in an increased surface area but also increased the active site density due to the formation of interfacial defects.^41^

In the previous sections, we summarized common strategies employed to exploit the HER potential of 2D MoSe_2_. Various experimental studies tested these concepts and reported performance enhancements. Table S2 summarizes the performance parameters and figures of merits for selected MoSe_2_-based electrocatalysts for the HER.

## 4 MoSe_2_ in Photocatalytic Hydrogen Generation

**Figure 5:**
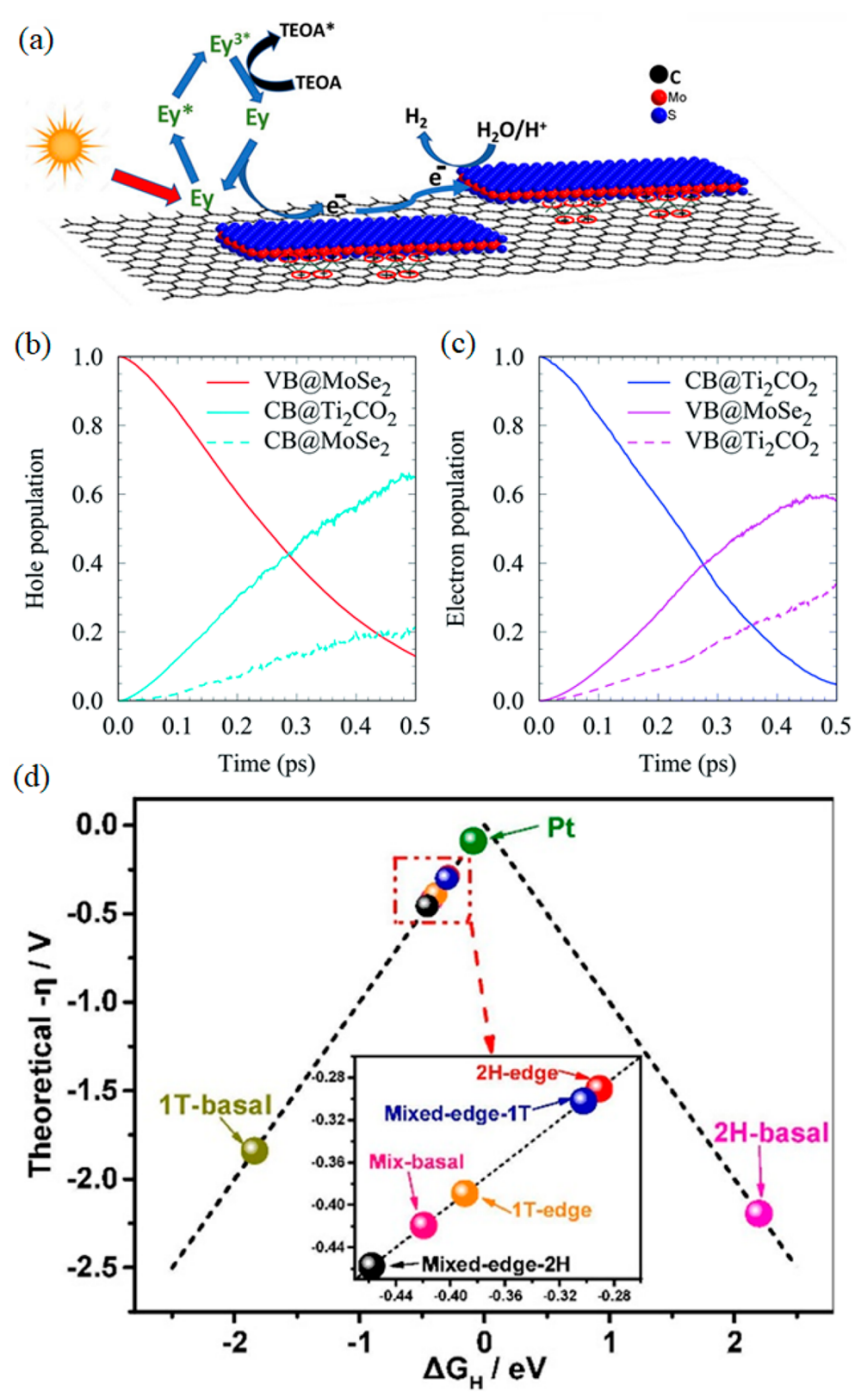
MoSe_2_ as cocatalyst for photocatalytic HER. (a) Dye-sensitized photocatalytic HER on a layered nanostructure of MoSe_2_. Reprinted with permission from ref (11). Copyright 2020 American Chemical Society. Time-dependent population of photogenerated (b) holes and (c) electrons in the CB and VB of MoSe_2_ and Ti_2_CO_2_. Reprinted with permission from ref (43). Copyright 2021 Royal Society of Chemistry. (d) Volcano-like correlation between the overpotential and the Gibbs free energy for hydrogen adsorption at different sites. Reprinted with permission from ref (47). Copyright 2020 Elsevier.

Although the conduction band minimum of MoSe_2_ is well above the water reduction potential, making it favorable for the HER, the small band gap of MoSe_2_ is not suitable for generation and separation of enough electron–hole pairs.^42^ In addition, inherent photocorrosion associated with 2D TMDCs also greatly limits the application of MoSe_2_ in photocatalytic H_2_ generation.^43^ Therefore, rather than developing MoSe_2_ as a standalone alternative to popular catalysts (e.g., TiO_2_) for photolysis, more focus was allocated to utilize MoSe_2_ in combination with other electron-generating materials as it provides active sites for H_2_ generation and acts as an efficient electron sink. Gupta et al.^42^ reported the photocatalytic HER activity of 1T-MoSe_2_ sensitized by Eosin Y dye. MoSe_2_ helps in the separation of the electron–hole pair as it acts as an electron sink and provides thermodynamically favorable active sites for hydrogen adsorption and evolution. Moreover, 1T-MoSe_2_ was reported to enhance the photocatalytic HER activity of g-C_3_N_4_ by 90 times by acting as a conduction band cocatalyst in 1T-MoSe_2_/g-C_3_N_4_.^44^ Similarly, MoSe_2_ acted as an electron sink and provided catalytic sites for H_2_ generation in hierarchical ZnIn_2_S_4_/MoSe_2_ nanoarchitectures to enhance the photocatalytic HER performance.^45^ Zeng et al.^46^ constructed a heterostructure of flower-like and network-like 2H MoSe_2_ with porous g-C_3_N_4_ to elucidate the dominant role of the active sites provided by MoSe_2_. The network-like MoSe_2_ exhibited superior HER performance owing to the synergistic effect of the sheet-on-sheet heterointerface, which effectively assists in charge separation and migration. Similar effects were also observed in layered nanocomposites of 2D MoSe_2_ with borocarbonitride and polymer-functionalized rGO (Figure 5a).^11^ Therefore, we believe that more efforts directed toward addressing poor charge separation and photocorrosion of MoSe_2_ could produce promising results.

Carefully aligning the band gap of MoSe_2_ with other semiconductors to construct a Z-scheme heterojunction is a promising strategy for ameliorating the detrimental electron–hole pair recombination. In addition, a Z-scheme catalyst can also extend the application of MoSe_2_ to total water splitting in a bicatalytic system by providing holes for the oxygen evolution reaction (OER). Moreover, fast transfer of photogenerated holes across the heterojunction can help remediate the inherent photocorrosion of MoSe_2_ and significantly enhance the stability of the catalytic system. Fu et al.^43^ proposed a 2D van der Waals (vdW) MoSe_2_/Ti_2_CO_2_ heterojunction for overall water splitting where Ti_2_CO_2_ and MoSe_2_ act as O_2_ and H_2_ evolution photocatalysts, respectively. The first-principles calculations revealed that the MoSe_2_/Ti_2_CO_2_ heterojunction resists photocorrosion and electron–hole recombination. Nonadiabatic molecular dynamics (NAMD) simulations predicted the ultrafast transfer of charge carriers across the heterojunction (Figure 5b and 5c). Notably, both the instant transfer of 65% holes from the VB of MoSe_2_ to the CB of Ti_2_CO_2_ and the antiphotocorrosion property of Ti_2_CO_2_ aided to the photostability of the heterojunction. Moreover, the unique band alignment of the heterojunction leads to a 12% theoretical solar-to-hydrogen (STH) energy conversion efficiency, making the Z-scheme MoSe_2_/Ti_2_CO_2_ photocatalyst promising for commercial application.

Mechanistically, a heterogeneous photocatalytic HER on the surface of a catalyst is like electrocatalytic H_2_ generation with an added complexity of electron–hole pair generation through light irradiation, their separation, and propagation to reaction sites. Therefore, strategies employed to enhance the electrocatalytic HER performance of MoSe_2_ can be improvised to assist in photocatalytic H_2_ generation. In addition, activating the basal planes and inactive edges and optimizing the H^+^ adsorption sites will improve the ability of MoSe_2_ to effectively receive and export the electron to H_2_ evolution sites. In that regard, multiphase MoSe_2_ was successfully incorporated in heterostructures with improved ability to receive electrons, higher HER activity, and improved stability.^47^ The comparison of H_2_ evolution activity at different active sites is illustrated in a volcano-like plot in Figure 5d. Moreover, the photoelectrochemical HER is another avenue to explore and can benefit from the synergy of the electro- and photocatalytic HER in a single catalyst. For instance, rhenium-doped MoSe_2_ exhibited enhanced photocurrent response.^48^Table S3 presents the performance evaluation of MoSe_2_-based photocatalysts for H_2_ generation.

## 5 Conclusions and Future Perspectives

MoSe_2_ is an excellent electrocatalyst for the HER owing to its low Gibbs free energy for hydrogen adsorption, narrow band gap, and more metallic nature. Nevertheless, the relatively low electrical conductivity, inactive basal planes, and aggregation of nanosheets during synthesis limit the application of pristine MoSe_2_. Fortunately, the physicochemical and electronic properties of MoSe_2_ can easily be tailored to suit the HER by simple techniques. Researchers have successfully realized desirable effects such as an increased number of active sites, improved electrical conductivity, stability, and enhanced morphology sufficiently exposing active sites in 2D MoSe_2_. Simple strategies including phase transition, defect engineering, heteroatom doping, and heterostructure formation are effective in enhancing the HER performance. For instance, the 2H → 1T phase transition of 2D MoSe_2_ enhances the electrical conductivity and activates the basal planes to improve the HER performance. Moreover, defect engineering and heteroatom doping can activate the basal planes and improve the conductivity without compromising the stability. We believe that combining multiple strategies in a single catalyst can result in maximized performance.

Nanostructure design is also an important tool to fully utilize the superior HER potential of 2D MoSe_2_. The formation of nanosheets and nanoflakes with enhanced morphology and exposed active sites boosts the overall performance. In that regard, sufficiently open nanoarchitectures, closely packing enough active sites, with interconnected electrical networks are desirable. This is often achieved using an interconnected network of highly conductive supports as the substrate for MoSe_2_ growth. Carbon-based materials are better suited as support materials owing to the good conductivity and ability to form interconnected networks. In addition, carbonaceous materials may also be activated for the HER by N doping. However, synthesis of a grid-like structure of MoSe_2_ is still challenging, which can provide superior conductivity and enhanced pathways for electrolyte and H_2_ diffusion. Moreover, the green synthesis of MoSe_2_-based catalysts should be pursued to make the H_2_ generation wholly green.

Combining MoSe_2_ with other active materials is another noteworthy technique to enhance the catalytic activity. The formation of a heterostructure results in an increased number of active sites, improved morphology, and amplified conductivity to synergistically enhance the electrocatalytic H_2_ generation. Moreover, optimized integration of multiple modification techniques in a single catalyst should be explored to further enhance the HER performance. However, identification of the best suited candidates is still dependent on human intuition, heuristics, and trial and error methods, which consume a lot of time and precious resources. Data-driven studies employing machine-learning models guided by ab initio calculations can accelerate the discovery of the best HER catalysts. The physicochemical properties of different combinations of materials and modulations can be predicted through theoretical calculations as well as data-driven approaches. Machine learning can further be helpful in screening suitable candidates from an array of potential materials. Finally, results can be verified through experimental studies. More information on the utilization of machine learning for material discovery can be found in recent reviews.^49,50^

MoSe_2_ also acts as good cocatalyst in the photocatalytic and photoelectrocatalytic HER by providing active sites and ameliorating electron–hole recombination by acting as an electron sink. The activity of MoSe_2_ as an electron exporter in photocatalytic H_2_ generation can be further enhanced through phase transition, defect engineering, doping, and other techniques employed for the electrocatalytic HER. Moreover, construction and experimental investigation of a direct Z-scheme photocatalyst of MoSe_2_ is a novel avenue to explore and might produce promising results for overall water splitting.

The application of 2D MoSe_2_ for other relevant applications such as the electrochemical nitrogen reduction reaction (NRR) to produce ammonia (NH_3_) should also be investigated. Similar to other TMDCs, 2D MoSe_2_ might also exhibit an enhanced ammonia yield rate as the spontaneous adsorption of N_2_ on the 1T phase of MoSe_2_ has already been established.^51^ Recently, Chen et al.^52^ effectively reduced N_2_ to NH_3_ with a high Faradaic efficiency of 37.82% by isolating single Au atoms onto MoSe_2._ We believe that the application of the above-mentioned strategies can also be effectively extended to enhance the performance of MoSe_2_-based catalysts for green NH_3_ production.

## Supporting Information Available

Gibbs free energy at different active sites in MoSe_2_; performance evaluation of various MoSe_2_-based electrocatalysts and photocatalysts (PDF)
