# Supplementary material for: Review on 2D Molybdenum Diselenide (MoSe2) and Its Hybrids for Green Hydrogen (H2) Generation Applications
Source: ACS Omega. 2022 May 12;7(20):16856–65. doi: 10.1021/acsomega.2c00330 (PMC9134225; doi:10.1021/acsomega.2c00330)
Supplement: Supplementary file 1 — ao2c00330_si_001.pdf [file ao2c00330_si_001.pdf]

Supplementary Information

A review on 2D Molybdenum Diselenide ($MoSe_2$) and their Hybrids for Green Hydrogen (H_2) Generation Applications

Muhammad B. Wazir [a], Muhammad Daud [b], Soma Safeer [b], Faisal AlMarzooqi [a], Ahsanulhaq Qurashi [c,*],

[a] Department of Chemical Engineering, Khalifa University of Science and Technology, Main Campus, 127788 Abu Dhabi, United Arab Emirates.

[b] Department of Chemical Engineering, University of Engineering and Technology, 25120 Peshawar, Pakistan.

[c] Department of Chemistry, Khalifa University of Science and Technology, Main Campus, Abu Dhabi, United Arab Emirates.

* Corresponding Author: Ahsanulhaq Qurashi

Email: ahsan.qurashi@ku.ac.ae

Table S1. Gibbs free energy at Mo and Se sites for different cases of vacancies. Reproduced with permissions from Ref. [1]. Copyrights (2018) American Chemical Society.

**Case**	**ΔG_{H^*} for H_{ads} at Mo sites (eV)**	**ΔG_{H^*} for H_{ads} at Se sites (eV)**
$MoSe_2$	2.11	1.93
V_{Mo}	-0.02	-0.11
V_{Se}	-0.16	1.78
V_{Se2}	-0.15	1.66
V_{MoSe2}	0.86	0.36

Table S2. Performance evaluation of $MoSe_2$ based catalysts for electrocatalytic HER.

Catalyst	Electrolyte	Surface area ($m^2\ g^{-1}$)	Current density (mA cm^{-2})	HER		Stability	Reference
				Over potential (mV)	Tafel slope (mV dec^{-1})		
Dry leaf-like mesoporous $MoSe_2$	0.5 M H_2SO_4	64.63	10	0.135 V	58	1000 CV cycles Slight degradation	2
1T-$MoSe_2$ $MoSe_2$-4-180 ($MoSe_2$-x-T, x= $NaBH_4$: $Na_2MoO_4 \cdot 2H_2O$, T:temp)	0.5 M H_2SO_4		10	152	52		3
2H/1T-$MoSe_2$ nanoflowers	0.5 M H_2SO_4	-	10	200	62 **TOF**:0.12 s^{-1}	5000 c No change	4
Plasma Etched $MoSe_2$ (at 20W)	0.5 M H_2SO_4	41	10	148	51.6	10 000 c small deterioration	5
2H/1T-$MoSe_2$, MS-6 (1T-74.1%)	-	-	10	85	57.6	Slight degradation over 20h	6
Monodisperse single/few-layered $MoSe_2$ nanocrystals directly deposited onto gold foil ED 1 h P2	0.5M H_2SO_4		10	107.2	31.8	2000 c Little activity loss	7
Cu-doped $MoSe_2$ Nanostripes (solution Deposition)	0.5 M H_2SO_4 purged with pure N2		10	86	44	20 h excellent robustness	8
Ni-doped $MoSe_2/Ti_2NT_x$	1 M KOH		10	92	79.7	18 h ,Slight activity loss	9
N-doped $MoSe_2$	0.5 M N2-saturated H_2SO_4			Onset over potential -135	62	1000 c slight degradation	10
N-$MoSe_2$/VG) shell/core nanoflake arrays	0.5 M H_2SO_4	14.2	10	-98	49	no decay after 20,000 c	11

3D carbonized melamine foam (CMF)/$MoSe_2$	0.5 M H_2SO_4				49.3	3000 c Slight degradation	[12]
$MoSe_2$-rGO-M (20 % rGO) (Aerosol synthesis)	0.5 M H_2SO_4	9.5	10	0.21 V	57	1000 c good durability	[13]
SWCNTs/$MoSe_2$	0.5 M H_2SO_4		10	0.17 V	67		[14]
$MoSe_2$/NDC	1 M KOH	220	10	155	68	10,000 c Slight degradation	[15]
$MoSe_2$/graphene/NF	0.5 M H_2SO_4		10	92	42	18h no activity loss	[16]
FL-$MoSe_2$/NC	0.5 M H_2SO_4	104.2	10	74.6	45.2 TOF: 5.7s-1@200mV	40,000 s No degradation	[17]
1T/2H $MoSe_2$/MXene	1M KOH (pH =14)		10	95	91	3000 c No activity loss	[18]
2D $MoSe_2$/CoP intercalated nanosheets	0.5 M H_2SO_4	98	10	105	51	1000 c negligible shift	[19]
CoP/$MoSe_2$-3 CoP:$MoSe_2$ = 3:1	0.5 M H_2SO_4		10	-135	67.5	1000 CV c ignorable difference	[20]
$MoSe_2$/MoO_2/Mo	0.5 M H_2SO_4		10	142	48.9	2000 c slight degradation	[21]
$MoSe_2$-Mo_2C	0.5 M H_2SO_4	8.9	10	73	51	20 h stable properties	[22]
$MoSe_2$/MoS_2	0.5 M H_2SO_4		10	162	61	2000 c almost no degradation	[23]
0D−2D $CoSe_2$/$MoSe_2$	1M KOH		10	90	84.8		[24]
Mo_2C/$MoSe_2$/Mo	1M KOH		10	51	47.6	5000 c ignorable difference	[25]
3D $MoSe_2$@$Ni_{0.85}$Se	1M KOH		10	117	66	20 h long-term stability during	[26]
Mo-Ni-Se@NF	1M KOH		10	113	85.7	20 h ignorable difference	[27]
$MoSe_2$/Rh	0.5 M H_2SO_4	61.5	10	0.192 V	47	1000 c	[28]

						little decrease	
Si-doped TiO_2 NTs/$MoSe_2$	0.5 M H_2SO_4	844	10	-94	40		[29]
1T-$MoSe_2$/Mo core-shell nanoscrews	0.5M H_2SO_4		10	166	34.7	1000 c<br>Slight degradation	[30]

Table S3. Performance evaluation of $MoSe_2$ based catalysts for photocatalytic HER.

**Catalyst**	**Notes**	**Surface area (m^2 g^{-1})**	**Sacrificial agent**	**Light source**	**Power of the lamp (W)**	**H_2 (mmol h^{-1} g^{-1})**	**Reference**
1 T-$MoSe_2$	Eosin Y<br>dye sanitized	-	Triethanolamine<br>(TEOA)	Visible light	100	62 ± 5<br>**TOF:** 15.5 ± 2 h^{-1}	[31]
$MoSe_2$/g-C_3N_4<br>(5wt% $MoSe_2$)	5N-CN<br>(network like-$MoSe_2$/g-C_3N_4)	62.2	10 vol% Triethanolamine	UV-cutoff filter ($\lambda > 420$ nm)	300	136.8 µmol g^{-1} h^{-1}	[32]
P.RGO/$MoSe_2$	1:9<br>(EY dye as the photosensitizer)	-	TEOA [20% (v/v)]	Visible light	400	9705 µmol g^{-1} h^{-1}	[33]
RGO/$MoSe_2$:Cu	-	301	-	UV light	-	85.5	[34]
WO3-CNT<br>@MoSe2<br>(4% MoSe2)	-	225	0.1 M Na_2S and<br>0.04 M Na_2SO_3	$\lambda \geq 400$ nm	-	161 µmol g^{-1} h^{-1}	[35]
Er^{3+}:$YAlO_3$/<br>Ta_2O_5-<br>$CaIn_2S_4$/$MoSe_2$-<br>RGO	MoSe2-RGO as co-catalyst<br>(1 wt %)	-	Methanol-water<br>(10.0 wt %)	($400 < \lambda < 800$ nm)	300	1050 µmol g^{-1}<br>(within 5.0 h)	[36]
Er^{3+}:$YAlO_3$/Ta_2O_5<br>/rGO/$MoSe_2$	mass ratio<br>Ta_2O_5:$MoSe_2$/r<br>GO =<br>99.25:0.75	-	Methanol-water<br>(10.0 wt %,pH = 6.00)	($400 < \lambda < 800$ nm)	300	182 µmol	[37]
(1T/2H)$MoSe_2$/C<br>dS-CdSe<br>MCCS-10	Hydrazine monohydrate as reductant	-	-	420 nm cut off<br>Filter	300	24.34	[38]

$WO_3/MoSe_2$@ carbon fiber		582.89	0.04 M Na_2SO_3 and 0.1 M Na_2S	**λ** ≥ 400 nm metal halide lamp	-	440	[39]
$ZnIn_2S_4$/2%$MoSe_2$	-	-	0.35M Na_2S and 0.25M Na_2SO_3	UV-cutoff filter (**λ** > 420 nm)	300	2228 µmol g^{-1} h^{-1}	[40]
$MoSe_2$-bridged MoS_2/CdS	6.0 wt.% FMS and 4.0 wt.% FMSe	-	20 vol.% aqueous lactic acid	Simulated solar light	150	193 µmol h^{-1}	[41]
2D $MoSe_2$/ TiO_2	0.1 wt.% $MoSe_2$	-	methanol	visible-light	300	5.13 µmol h^{-1}	[42]
1T/2H-$MoSe_2$/ 1D-CdS	1T/2H-MC-10% $NaBH_4$ as reductant	23.69	10 vol% of lactic acid	λ > 420 nm	300	93.4 mmol g^{-1} h^{-1}	[43]
($MoSe_2$-RGO)/(Er^{3+}:Y3Al_5O_{12}/ZnS)/RuO_2		-	0.20 mol/L Na_2S and 0.30 mol/L Na_2SO_3	420 < λ < 760 nm	300	302.4 µmol/g (in 5h)	[44]

References:

(1) Xia, B.; Wang, T.; Jiang, X.; Zhang, T.; Li, J.; Xiao, W.; Xi, P.; Gao, D.; Xue, D.; Ding, J. Ar^{2+} Beam Irradiation-Induced Multivancancies in $MoSe_2$ Nanosheet for Enhanced Electrochemical Hydrogen Evolution. *ACS Energy Lett.* **2018**, *3* (9), 2167–2172.

(2) Vattikuti, S. V. P.; Devarayapalli, K. C.; Nagajyothi, P. C.; Shim, J. Microwave Synthesized Dry Leaf-like Mesoporous $MoSe_2$ Nanostructure as an Efficient Catalyst for Enhanced Hydrogen Evolution and Supercapacitor Applications. *Microchem. J.* **2020**, *153*, 104446.

(3) Yin, Y.; Zhang, Y.; Gao, T.; Yao, T.; Zhang, X.; Han, J.; Wang, X.; Zhang, Z.; Xu, P.; Zhang, P.; Cao, X.; Song, B.; Jin, S. Synergistic Phase and Disorder Engineering in 1T-$MoSe_2$ Nanosheets for Enhanced Hydrogen-Evolution Reaction. *Adv. Mater.* **2017**, *29* (28), 1700311.

(4) Wu, J.; Li, B.; Shao, Y.; Wu, X.; Sun, Y. Tuning the Morphology and Phase of $MoSe_2$ by Using a Mixed Solvent of Water and Dimethyl Formamide and Its Enhanced Electrocatalytic Activity for Hydrogen Evolution Reaction. *J. Mater. Sci.* **2020**, *55* (5), 2129–2138.

(5) Xiao, D.; Ruan, Q.; Bao, D.; Luo, Y.; Huang, C.; Tang, S.; Shen, J.; Cheng, C.; Chu, P. K. Effects of Ion Energy and Density on the Plasma Etching-Induced Surface Area, Edge Electrical Field, and Multivacancies in $MoSe_2$ Nanosheets for Enhancement of the Hydrogen Evolution Reaction. *Small* **2020**, *16* (25), 2001470.

(6) Xiao, W.; Bukhvalov, D.; Zou, Z.; Zhang, L.; Lin, Z.; Yang, X. Unveiling the Origin of the High Catalytic Activity of Ultrathin 1T/2H $MoSe_2$ Nanosheets for the Hydrogen Evolution Reaction: A Combined Experimental and Theoretical Study. *ChemSusChem* **2019**, *12* (22), 5015–5022.

(7) Damien, D.; Anil, A.; Chatterjee, D.; Shaijumon, M. M. Direct Deposition of $MoSe_2$ Nanocrystals onto Conducting Substrates: Towards Ultra-Efficient Electrocatalysts for Hydrogen Evolution. *J. Mater. Chem. A* **2017**, *5* (26), 13364–13372.

(8) Vikraman, D.; Hussain, S.; Akbar, K.; Karuppasamy, K.; Chun, S.-H.; Jung, J.; Kim, H.-S. Design of Basal Plane Edges in Metal-Doped Nanostripes-Structured $MoSe_2$ Atomic Layers To Enhance Hydrogen Evolution Reaction Activity. *ACS Sustain. Chem. Eng.* **2019**, *7* (1), 458–469.

(9) Zong, H.; Yu, K.; Zhu, Z. Heterostructure Nanohybrids of Ni-Doped $MoSe_2$ Coupled with Ti_2NTx toward Efficient Overall Water Splitting. *Electrochim. Acta* **2020**, *353*, 136598.

(10) Ren, X.; Ma, Q.; Ren, P.; Wang, Y. Synthesis of Nitrogen-Doped $MoSe_2$ Nanosheets with Enhanced Electrocatalytic Activity for Hydrogen Evolution Reaction. *Int. J. Hydrogen Energy* **2018**, *43* (32), 15275–15280.

(11) Deng, S.; Zhong, Y.; Zeng, Y.; Wang, Y.; Yao, Z.; Yang, F.; Lin, S.; Wang, X.; Lu, X.; Xia, X.; Tu, J. Directional Construction of Vertical Nitrogen-Doped 1T-2H $MoSe_2$ /Graphene Shell/Core Nanoflake Arrays for Efficient Hydrogen Evolution Reaction. *Adv. Mater.* **2017**, *29* (21), 1700748.

(12) Huang, Y.; Cui, F.; Zhao, Y.; Lian, J.; Bao, J.; Liu, T.; Li, H. 3D Hierarchical CMF/$MoSe_2$ Composite Foam as Highly Efficient Electrocatalyst for Hydrogen Evolution. *Electrochim. Acta* **2018**, *263*, 94–101.

(13) Park, S.-K.; Park, G. D.; Ko, D.; Kang, Y. C.; Piao, Y. Aerosol Synthesis of Molybdenum Diselenide–Reduced Graphene Oxide Composite with Empty Nanovoids and Enhanced Hydrogen Evolution Reaction Performances. *Chem. Eng. J.* **2017**, *315*, 355–363.

(14) Najafi, L.; Bellani, S.; Oropesa-Nuñez, R.; Ansaldo, A.; Prato, M.; Del Rio Castillo, A. E.; Bonaccorso, F. Engineered $MoSe_2$ -Based Heterostructures for Efficient Electrochemical Hydrogen Evolution Reaction. *Adv. Energy Mater.* **2018**, *8* (16), 1703212.

(15) Cao, Z.; Hu, H.; Wu, M.; Tu, C.; Zhang, D.; Wu, Z. Confined Growth of $MoSe_2$ Nanosheets in N-Doped Carbon Shell with Hierarchical Porous Structure for Efficient Hydrogen Evolution. *Sustain. Energy Fuels* **2019**, *3* (9), 2409–2416.

(16) Hussain, S.; Vikraman, D.; Akbar, K.; Naqvi, B. A.; Abbas, S. M.; Kim, H.-S.; Chun, S.-H.; Jung, J. Fabrication of $MoSe_2$ Decorated Three-Dimensional Graphene Composites Structure as a Highly Stable Electrocatalyst for Improved Hydrogen Evolution Reaction. *Renew. Energy* **2019**, *143*, 1659–1669.

(17) Jiang, Z.-J.; Xie, G.; Deng, B.; Jiang, Z. More Active Sites Exposed Few-Layer $MoSe_2$ Supported on Nitrogen-Doped Carbon as Highly Efficient and Durable Electrocatalysts for Water Splitting. *Electrochim. Acta* **2018**, *285*, 103–110.

(18) Li, N.; Zhang, Y.; Jia, M.; Lv, X.; Li, X.; Li, R.; Ding, X.; Zheng, Y.-Z.; Tao, X. 1T/2H $MoSe_2$-on-MXene Heterostructure as Bifunctional Electrocatalyst for Efficient Overall Water Splitting. *Electrochim. Acta* **2019**, *326*, 134976.

(19) Zhang, L.; Zhu, J.; Wang, Z.; Zhang, W. 2D $MoSe_2$/CoP Intercalated Nanosheets for Efficient Electrocatalytic Hydrogen Production. *Int. J. Hydrogen Energy* **2020**, *45* (38), 19246–19256.

(20) Ding, C.; Qian, J.; Li, Z.; Li, Y.; Peng, W.; Zhang, G.; Zhang, F.; Fan, X. Cobalt Phosphide Nanoparticles Anchored on Molybdenum Selenide Nanosheets as High-Performance Electrocatalysts for Water Reduction. *Int. J. Hydrogen Energy* **2018**, *43* (45), 20346–20353.

(21) Jian, C.; Cai, Q.; Hong, W.; Li, J.; Liu, W. Edge-Riched $MoSe_2$/MoO_2 Hybrid Electrocatalyst for Efficient Hydrogen Evolution Reaction. *Small* **2018**, *14* (13), 1703798.

(22) Vikraman, D.; Hussain, S.; Karuppasamy, K.; Feroze, A.; Kathalingam, A.; Sanmugam, A.; Chun, S.-H.; Jung, J.; Kim, H.-S. Engineering the Novel $MoSe_2$-Mo_2C Hybrid Nanoarray Electrodes for Energy Storage and Water Splitting Applications. *Appl. Catal. B Environ.* **2020**, *264*, 118531.

(23) Li, S.; Zang, W.; Liu, X.; Pennycook, S. J.; Kou, Z.; Yang, C.; Guan, C.; Wang, J. Heterojunction Engineering of $MoSe_2$/MoS_2 with Electronic Modulation towards Synergetic Hydrogen Evolution Reaction and Supercapacitance Performance. *Chem. Eng. J.* **2019**, *359*, 1419–1426.

(24) Xia, L.; Song, H.; Li, X.; Zhang, X.; Gao, B.; Zheng, Y.; Huo, K.; Chu, P. K. Hierarchical 0D−2D Co/Mo Selenides as Superior Bifunctional Electrocatalysts for Overall Water Splitting. *Front. Chem.* **2020**, *8*.

(25) Li, J.; Hong, W.; Jian, C.; Cai, Q.; He, X.; Liu, W. High-Performance Hydrogen Evolution at a $MoSe_2$–Mo_2C Seamless Heterojunction Enabled by Efficient Charge Transfer. *J. Mater. Chem. A* **2020**, *8* (14), 6692–6698.

(26) Wang, C.; Zhang, P.; Lei, J.; Dong, W.; Wang, J. Integrated 3D $MoSe_2$@$Ni_{0.85}Se$ Nanowire Network with Synergistic Cooperation as Highly Efficient Electrocatalysts for Hydrogen Evolution Reaction in Alkaline Medium. *Electrochim. Acta* **2017**, *246*, 712–719.

(27) Yang, H.; Huang, Y.; Teoh, W. Y.; Jiang, L.; Chen, W.; Zhang, L.; Yan, J. Molybdenum Selenide Nanosheets Surrounding Nickel Selenides Sub-Microislands on Nickel Foam as High-Performance Bifunctional Electrocatalysts for Water Splitting. *Electrochim. Acta* **2020**, *349*, 136336.

(28) Sharma, M. D.; Mahala, C.; Basu, M. Nanosheets of $MoSe_2$@M (M = Pd and Rh) Function as Widespread PH Tolerable Hydrogen Evolution Catalyst. *J. Colloid Interface Sci.* **2019**, *534*, 131–141.

(29) He, H.-Y.; He, Z.; Shen, Q. TiO 2 : Si Nanotube/1T-$MoSe_2$ Nanosheet Hybrids with Highly Efficient Hydrogen Evolution Catalytic Activity. *J. Colloid Interface Sci.* **2018**, *522*, 136–143.

(30) Qu, Y.; Medina, H.; Wang, S.-W.; Wang, Y.-C.; Chen, C.-W.; Su, T.-Y.; Manikandan, A.; Wang, K.; Shih, Y.-C.; Chang, J.-W.; Kuo, H.-C.; Lee, C.-Y.; Lu, S.-Y.; Shen, G.; Wang, Z. M.; Chueh, Y.-L. Wafer Scale Phase-Engineered 1T- and 2H-$MoSe_2$/Mo Core-Shell 3D-Hierarchical Nanostructures toward Efficient Electrocatalytic Hydrogen Evolution Reaction. *Adv. Mater.* **2016**, *28* (44), 9831–9838.

(31) Gupta, U.; Naidu, B. S.; Maitra, U.; Singh, A.; Shirodkar, S. N.; Waghmare, U. V.; Rao, C. N. R. Characterization of Few-Layer 1T-$MoSe_2$ and Its Superior Performance in the Visible-Light Induced Hydrogen Evolution Reaction. *APL Mater.* **2014**, *2* (9), 092802.

(32) Zeng, D.; Wu, P.; Ong, W.-J.; Tang, B.; Wu, M.; Zheng, H.; Chen, Y.; Peng, D.-L. Construction of Network-like and Flower-like 2H-$MoSe_2$ Nanostructures Coupled with Porous g-C_3N_4 for Noble-Metal-Free Photocatalytic H2 Evolution under Visible Light. *Appl. Catal. B Environ.* **2018**, *233*, 26–34.

(33) Pramoda, K.; Servottam, S.; Kaur, M.; Rao, C. N. R. Layered Nanocomposites of Polymer-Functionalized Reduced Graphene Oxide and Borocarbonitride with MoS_2 and $MoSe_2$ and Their Hydrogen Evolution Reaction Activity. *ACS Appl. Nano Mater.* **2020**, *3* (2), 1792–1799.

(34) He, H.-Y.; He, Z.; Shen, Q. Reduced Graphene Oxide/Metallic $MoSe_2$: Cu Nanosheet Nanostructures Grown by a Chemical Process for Highly Efficient Water Splitting. *Mater. Res. Bull.* **2019**, *111*, 183–190.

(35) Tahir, M. B.; Nabi, G.; Iqbal, T.; Sagir, M.; Rafique, M. Role of $MoSe_2$ on Nanostructures WO_3-CNT Performance for Photocatalytic Hydrogen Evolution. *Ceram. Int.* **2018**, *44* (6), 6686–6690.

(36) Wei, S.; Song, Y.; Rong, Y.; Tang, L.; Chen, Y.; Lu, C.; Zhang, Z.; Wang, J. A Novel Z-Scheme Er3+:$YAlO_3$/Ta_2O_5-$CaIn_2S_4$/$MoSe_2$-Reduced Graphene Oxide Photocatalyst with Superior Photocatalytic Hydrogen Evolution Activity. *Renew. Energy* **2017**, *111*, 628–

637.
(37) Wang, G.; Ma, X.; Wang, C.; Li, S.; Qiao, J.; Zhang, H.; Li, G.; Wang, J.; Song, Y. Highly Efficient Visible-Light Driven Photocatalytic Hydrogen Evolution over Er^{3+}:$YAlO_3$/Ta_2O_5/RGO/$MoSe_2$ Nanocomposite. *J. Mol. Liq.* **2018**, *260*, 375–385.
(38) Liu, Y.; Li, Y.; Lin, Y.; Yang, S.; Zhang, Q.; Peng, F. Theoretical Calculations and Controllable Synthesis of $MoSe_2$/CdS-CdSe with Highly Active Sites for Photocatalytic Hydrogen Evolution. *Chem. Eng. J.* **2020**, *383*, 123133.
(39) Tahir, M. B.; Asiri, A. M.; Nabi, G.; Rafique, M.; Sagir, M. Fabrication of Heterogeneous Photocatalysts for Insight Role of Carbon Nanofibre in Hierarchical WO_3/MoS Composite for Enhanced Photocatalytic Hydrogen Generation. *Ceram. Int.* **2019**, *45* (5), 5547–5552.
(40) Zeng, D.; Xiao, L.; Ong, W.-J.; Wu, P.; Zheng, H.; Chen, Y.; Peng, D.-L. Hierarchical $ZnIn_2S_4$/$MoSe_2$ Nanoarchitectures for Efficient Noble-Metal-Free Photocatalytic Hydrogen Evolution under Visible Light. *ChemSusChem* **2017**, *10* (22), 4624–4631.
(41) Kumar, D. P.; Kim, E. H.; Park, H.; Chun, S. Y.; Gopannagari, M.; Bhavani, P.; Reddy, D. A.; Song, J. K.; Kim, T. K. Tuning Band Alignments and Charge-Transport Properties through $MoSe_2$ Bridging between MoS_2 and Cadmium Sulfide for Enhanced Hydrogen Production. *ACS Appl. Mater. Interfaces* **2018**, *10* (31), 26153–26161.
(42) Wu, L.; Shi, S.; Li, Q.; Zhang, X.; Cui, X. TiO_2 Nanoparticles Modified with 2D $MoSe_2$ for Enhanced Photocatalytic Activity on Hydrogen Evolution. *Int. J. Hydrogen Energy* **2019**, *44* (2), 720–728.
(43) Li, N.; Wu, J.; Lu, Y.; Zhao, Z.; Zhang, H.; Li, X.; Zheng, Y.-Z.; Tao, X. Stable Multiphasic 1T/2H $MoSe_2$ Nanosheets Integrated with 1D Sulfide Semiconductor for Drastically Enhanced Visible-Light Photocatalytic Hydrogen Evolution. *Appl. Catal. B Environ.* **2018**, *238*, 27–37.
(44) Li, S.; Liu, Z.; Lu, C.; Qu, Z.; Piao, C.; Tang, J.; Fang, D.; Wang, J. Highly Efficient Visible-Light Driven Photocatalytic Hydrogen Production over ($MoSe_2$-RGO)/(Er^{3+}:$Y_3Al_5O_{12}$/ZnS)/RuO_2 Photocatalyst. *J. Photochem. Photobiol. A Chem.* **2020**, *400*, 112714.
